# ACS6, a Hydrogen sulfide-donating derivative of sildenafil, inhibits homocysteine-induced apoptosis by preservation of mitochondrial function

**DOI:** 10.1186/2045-9912-1-20

**Published:** 2011-08-16

**Authors:** Xiao-Qing Tang, Rong-Qian Chen, Yan-Kai Ren, Piero Del Soldato, Anna Sparatore, Yuan-Yuan Zhuang, Hen-Rong Fang, Chun-Yan Wang

**Affiliations:** 1 Department of Physiology, Medical College, University of South China, Hengyang, 421001, Hunan, P.R. China; 2 CTG Pharma, Milano, 20131, Italy; 3 Department of Pharmaceutical Sciences "Pietro Pratesi", Università degli Studi di Milano, Milan, Italy; 4 Department of Pathophysiology, Medical College,, University of south China, Hengyang, 421001, Hunan, P.R. China

**Keywords:** H_2_S-releasing sildenafil, Apoptosis, Homocysteine, Mitochondrial membrane potential, Reactive oxygen species, Bcl-2

## Abstract

**Background:**

The hydrogen sulfide-releasing sildenafil, ACS6, has been demonstrated to inhibit superoxide formation through donating hydrogen sulfide (H_2_S). We have found that H_2_S antagonizes homocysteine-induced oxidative stress and neurotoxicity. The aim of the present study is to explore the protection of ACS6 against homocysteine-triggered cytotoxicity and apoptosis and the molecular mechanisms underlying in PC12 cells.

**Methods:**

Cell viability was determined by Cell Counting Kit-8 assay. Cell apoptosis was observed using the chromatin dye Hoechst 33258 and analyzed by Flow Cytometry after propidium iodide staining. Mitochondrial membrane potential was monitored using the fluorescent dye Rh123. Intracellular reactive oxygen species were determined by oxidative conversion of cell permeable 2',7'-dichlorfluorescein-diacetate to fluorescent 2',7'-dichlorfluorescein. The expression of cleaved caspase-3 and bcl-2 and the accumulation of cytosolic cytochrome *c *were analyzed by Western blot.

**Results:**

We show that ACS6 protects PC12 cells against cytotoxicity and apoptosis induced by homocysteine and blocks homocysteine-triggered cytochrome c release and caspase-3 activation. ACS6 treatment results in not only prevention of homocysteine-caused mitochondrial membrane potential (Δψ) loss and reactive oxygen species (ROS) overproduction but also reversal of Bcl-2 down-expression.

**Conclusions:**

These results indicate that ACS6 protects PC12 cells against homocysteine-induced cytotoxicity and apoptosis by preservation of mitochondrial function though inhibiting both loss of Δψ and accumulation of ROS as well as modulating the expression of Bcl-2. Our study provides evidence both for a neuroprotective effect of ACS6 and for further evaluation of ACS6 as novel neuroprotectants for Alzheimer's disease associated with homocysteine.

## Introduction

Homocysteine, a thiol-containing amino acid, is a key metabolic intermediate in sulfuramino acid metabolism [1,2]. Homocysteine not only can be remethylated to methionine by enzymes that require folic acid but also can be catabolized to form cysteine by cystathionine-β-synthetase (CBS). Both in vitro and in vivo studies have shown that homocysteine is toxic to neuronal cells [3-9]. One explanation for the mechanism of homocysteine neurotoxicity is that auto-oxidation of homocysteine leads to the formation of superoxide and hydrogen peroxide [10]. The causative link between hyperhomocysteinemia and neurodegeneration has been known [11]. Elevated brain homocysteine has been reported in Alzheimer's disease (AD) [12]. It is now established that elevated plasma homocysteine is a strong, independent risk factor of AD [13-17]. Therefore, the potential role of homocysteine is regarded as a novel therapeutic target for AD [17].

Hydrogen sulfide (H_2_S), a well-known toxic gas with the smell of rotten eggs, is formed naturally in mammalian tissues and exhibits a series of biological and physiological effects [18-21]. It has been recognized as an important endogenous neuromodulator [18,22]. In the central nervous system, endogenous H_2_S is synthesized from L-cysteine and this process is predominantly catalyzed by CBS [19,23]. The roles of H_2_S in neuroprotection have been extensively reported [21,24]. H_2_S protects primary rat cortical neurons from oxytosis induced by glutamate [25] as well as SHSY-5Y cells against the neurotoxicity of peroxynitite (ONOO**^¯^**) [26] and hypochlorous acid (HOCl) [27]. We also have reported that H_2_S produces neuroprotective effects when it is used to treat beta-amyloid- and 1-methyl-4-phenyl pyridium ion (MPP^+^)-induced neurotoxicity [28-30]. Most recent study by our group have demonstrated that H_2_S protects neurons from damage caused by homocysteine to neurons [31], suggesting a promising role of H_2_S supplement as a novel therapeutic strategy for AD associated with homocysteine.

The pharmacological profile of a new, safe and effective H_2_S-releasing sildenafil (ACS6) was described recently [32]. Muzaffar et al. reported that ACS6 is a potent inhibitor of superoxide formation and that H_2_S release from ACS6 is crucial for its biological actions [32]. Thus, it is logical to test the role of ACS6 in homocysteine-induced neurotoxicity.

The purpose of this study therefore is to investigate the effects of ACS6 on homocysteine-induced neurotoxicity to PC12 cells, a clonal rat pheochromocytoma cell line, which is widely used for studying the cellular biology of neurons (33-35). We demonstrated for the first time that ACS6, a putative H_2_S-donating derivative of sildenafil, significantly protected PC12 cells against homocysteine-induced cytotoxicity and apoptosis by inhibition of reactive oxygen species (ROS) accumulation, preservation of mitochondrial membrane potential (Δψ) and up-regulation of bcl-2 expression. Our findings suggest that ACS6, acting as an H_2_S donor, is able to act as a neuroprotectant.

## Materials and methods

### Materials

**Figure 1:**
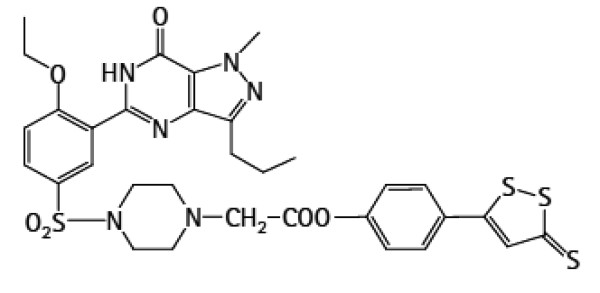
**The chemical structure of ACS6**.

Hoechst 33258, Rhodamine 123 (Rh123), 2',7'-dichlorfluorescein- diacetate (DCFH-DA) and homocysteine were purchased from Sigma Chemical CO (st.Louis, MO, USA). ACS6, 1-Piperazineacetic acid 4-[[3-(4,7-dihydro-1-methyl-7-oxo-3-propyl-1H-pyrazolo[4,3-d]pyrimidin-5-yl)-4- ethoxyphenyl]sulphonyl]-,4-(3-thioxo-3H-1,2-dithiol-5-yl)phenyl ester, was supplied by CTG Pharma, Milan, Italy (The chemical structure of ACS6 is shown in Figure 1). Cell counter kit-8 (CCK-8) was bought from Dojindo Lab. (Rockville, MD, USA). Antibodies for detecting bcl-2 and cleaved caspase-3 were obtained from Cell Signaling Technology, Inc (Beverly, MA, USA). Antibody against cytochrome c (Cyt-c) was purchased from Abcam Technology (Cambridge, CB, UK). RPMI-1640 medium, horse serum and fetal bovine serum were supplied by Gibico BRL (Ground Island, NY, USA).

### Cell culture

PC12 cells, a rat cell line derived from a Pheochromocytoma cells, were supplied from Sun Yat-sen University Experimental Animal Center (Guangzhou, China), and were maintained on tissue culture plastic in RPMI-1640 medium supplemented with 10% heat-inactivated horse serum and 5% fetal bovine serum (FBS) at 37°C under an atmosphere of 5% CO_2 _and 95% air. The culture media was changed three times per week.

### Determination of cell viability

The viability of PC12 cells was determined by Cell Counting Kit-8 (CCK-8) assay. PC12 cells were cultured in 96-well plates at 37°C under an atmosphere of 5% CO_2 _and 95% air. When the cells were about 70% fusion, indicated conditioned-mediums were administered. At the end of treatment, 10 μl CCK-8 solutions were added into each well and then the plates were incubated for 3 h in the incubator. Absorbance at 450 nm was measured with a microplate reader (Molecular Devices, Sunnyvale, CA, USA). Means of four wells optical density (OD) in the indicated groups were used to calculate the percentage of cell viability according to the formula below: cell viability (%) = OD treatment group/OD control group × 100%. The experiment was repeated three times.

### Nuclear staining for assessment of apoptosis

Chromosomal condensation and morphological changes in the nucleus of PC12 cells were observed using the chromatin dye Hoechst 33258. The PC12 cells were fixed with 4% paraformaldehyde in 0.1 M phosphate buffered saline (PBS) for 10 min. After three rinses with PBS, the cells were stained with 5 mg/L Hoechst 33258 for 10 min. Slides were rinsed briefly with PBS, air dried, then mounted in an anti-fluorescein fading medium (Perma Fluor, Immunon, PA, USA). Slides were visualized under a fluorescent microscope (BX50-FLA, Olympus, Tokyo, Japan). Viable cells displayed normal nuclear size and uniform fluorescence, whereas apoptotic cells showed condensed nuclei or nuclear condensations. The percentage of apoptotic cells was evaluated as follows: The percentage of apoptotic cells = The numbers of apoptotic cells/(The numbers of apoptotic cells + The numbers of viable cells) × 100%.

### Measurement of the mitochondrial membrane potential (Δψ)

Δψ was monitored using the fluorescent dye Rh123, a cell permeable cationic dye, which preferentially enters into mitochondria based on the highly negative Δψ. Depolarization of Δψ results in the loss of Rh123 from the mitochondria and a decrease in intracellular fluorescence [36]. Rh123 (100 μg/L) was added to cell cultures for 45 min at 37°C. Rh123 fluorescence was measured by flow cytometry (FCM, Beckman-coulter Co., USA). Ten thousand cells per sample were analyzed and the mean fluorescent intensity (MFI) in the positive cells represents the level of Δψ.

### Measurement of intracellular ROS generation

Intracellular ROS were determined by oxidative conversion of cell permeable 2',7'-dichlorfluorescein-diacetate (DCFH-DA) to fluorescent 2',7'-dichlorfluorescein (DCF) [37,38]. The cells were collected by pipetting and were washed one time with PBS. After DCFH-DA (2.5 *μ*M) was added to cell cultures for 20 min at 37°C, the cells were washed twice with PBS. The mean fluorescent intensity (MFI) of the positive cells in ten thousand cells per sample was measured by FCM, and the MFI represents the amount of ROS.

### Western blot analysis for cleaved caspase-3 and bcl-2

SDS-polyacrylamide gel electrophoresis (PAGE) was carried out on 5% stacking and 12% resolving gel with low range molecular weight standards (Solarbio, China). Equal amounts of protein were loaded in each lane with loading buffer (Beyotime, China) containing 0.1 M Tris (pH6.8), 20% glycerol, 10% mercaptoethanol, 4% SDS and 0.2% Bromophenol Blue. Samples were heated at 100°C for 5 min before gel loading. Following electrophoresis, the proteins were transferred to a PVDF transfer membrane (Solarbio, China). After this, the membranes were blocked with TBST (50 mM Tris-HCl, pH 7.4, 0.15 M NaCl, 0.1% Tween-20) containing 5% BSA (Sigma, USA) for 2 h. Following this, the membranes were incubated with primary antibodies diluted 1:1000 at 4°C over night. After washing with TBST, the membranes were incubated with anti-rabbit IgG labeled with horseradish peroxidase (Zsbio, China) diluted at 1:1000 at room temperature for 2 h. The membranes were washed again and developed with an enhanced chemiluminescence system (ECL, Zsbio, China) followed by apposition of the membranes with autoradiographic films (Kodak, China). The integrated optical density for the protein band was calculated by Image-J software.

### Analysis of Cytosolic Cytochrome *c *Accumulation

Cytochrome *c *release from mitochondria into the cytosol was measured by Western blot analysis. The cells were collected by centrifugation at 200*g *for 10 min at 4°C. The pellets were then washed twice with chilled PBS and added with 400 μl of lysis buffer containing 250 mM sucrose, 20 mM HEPES-KOH, pH 7.4, 10 mM KCl, 1.5 mM Na-EGTA, 1.5 mM Na-EDTA, 1 mM MgCl2, 1 mM dithiothreitol, and a cocktail of protease inhibitors (Roche Diagnostics, Shanghai, China). After incubation on ice for 5 min, the cells were gently scraped off and centrifuged at 1000*g *for 10 min at 4°C. The supernatants were further centrifuged at 16,000*g *for 25 min at 4°C. The resulting supernatant was used as the soluble cytosolic fraction and subjected to Western blot analysis as mentioned above.

### Statistical analysis

Data are expressed as mean ± SEM. The significance of inter-group differences was evaluated by one-way analyses of variance (ANOVA: Least-significant difference's test for post hoc comparisons). Differences were considered significant at *P *< 0.05.

## Results

### ACS6 suppresses homocysteine-induced cytotoxicity

**Figure 2:**
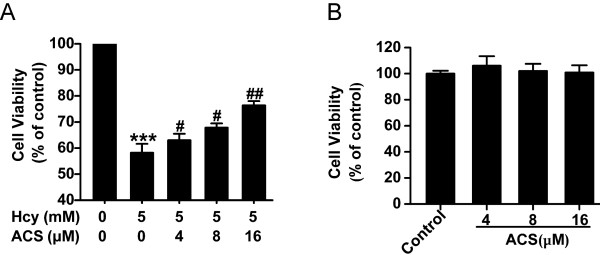
**ACS6 protects PC12 cells against homocysteine-induced cytotoxicity**. (A) PC12 cells were pretreated with ACS6 (4, 8, or 16 μmol/L) for 30 min and then exposed to homocysteine (Hcy, 5 mmol/L) for 24 h. (B) PC12 cells were treated with 4, 8, and 16 μmol/L ACS6 for 24 h. Cell viability was determined by CCK-8 assay. Values are the mean ± SEM (*n *= 3). ***P < 0.001, versus control group; ^#^*P *< 0.05, ^##^*P *< 0.01, versus 5 mmol/L homocysteine-treated alone group.

To investigate the effect of ACS6 on homocysteine-induced cytotoxicity, cell viability was analyzed by CCK-8 assay. As shown in Figure 2A, treatment with homocysteine (5 mmol/L) for 24 h significantly attenuated cell viability and the cytotoxic effect of homocysteine on PC12 cells was blocked by pretreatment with ACS6 at the concentrations of 4, 8, and 16 μmol/L for 30 min in a concentration-dependent manner. ACS6 (from 4 μmol/L to 16 μmol/L) alone did not measurably affect the viability of PC12 cells (Figure 2B). These results indicate that ACS6 protects PC12 cells against homocysteine-caused cytotoxicity.

### ACS6 inhibits homocysteine-induced apoptosis

**Figure 3:**
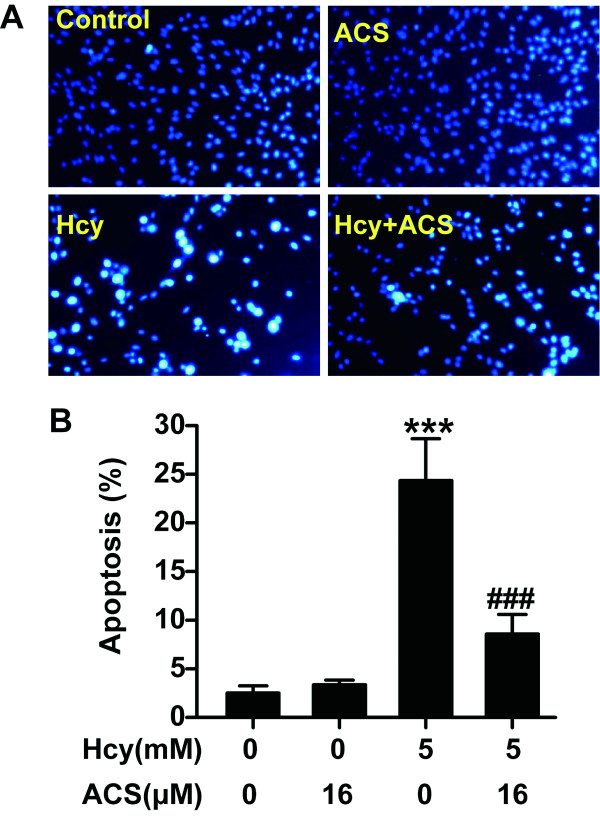
**Nuclear staining to evaluate the anti-apoptotic effect of ACS6**. After pretreated with 16 μmol/L ACS6 for 30 min, PC12 cells were exposed to 5 mmol/L homocysteine (Hcy) for 24 h and incubated with 5 mg/L Hoechst 33258 for 30 min. (A) Representative morphology visualized under a fluorescence microscope (10 × objective, BX50-FLA, Olympus). Cells with brightly fluorescent and fragmented nuclei were apoptotic. (B) Quantitative analysis of the percentage of apoptotic cells. Values are the mean ± SEM (*n *= 5). ***P < 0.001, versus control group; ^###^*P *< 0.001, versus 5 mmol/L homocysteine-treated alone group.

The nuclear staining assay was used to assess the morphological changes of apoptosis in PC12 cells. As illustrated in Figure 3, the untreated cells and the cells treated with 16 μmol/L ACS exhibited uniformly dispersed chromatin and intact cell membrane. On the other hand, the homocysteine-treated cells (5 mmol/L, for 24 h) appeared typical characteristics of apoptosis, including apoptotic nuclear condensation. When PC12 cells were pretreated with 16 μmol/L ACS6, however, the number of cells with nuclear condensation induced by 24 h exposure to 5 mmol/L homocysteine was significantly reduced, suggesting that ACS6 protects PC12 cells against apoptosis induced by homocysteine.

### ACS6 decreases homocysteine-induced release of Cyt-c

**Figure 4:**
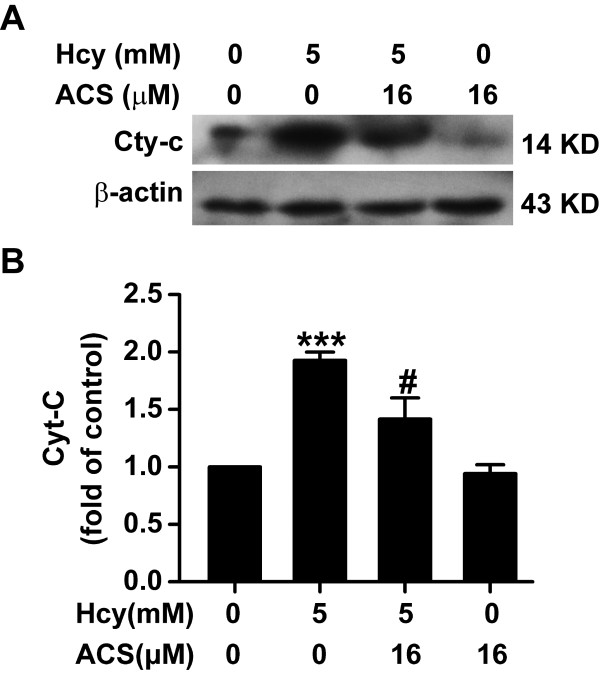
**Effects of ACS6 on the release of Cyt-c in PC12 cells**. After pretreated with 16 μmol/L ACS6 for 30 min, PC12 cells were exposed to 5 mmol/L homocysteine (Hcy) for 24 h. A, representative immunoblots for Cyt-c release from three independent experiments. Cytosolic fractions of the extract were subjected to Western blot analysis using an anti-Cyt-c antibody. In all blots, staining for β-actin was used as a loading control. B, quantification of cytosolic Cyt-c accumulation as a percent of the control. Values are the mean ± SEM (*n *= 3). ***P <0.001, versus control; ^#^*P *< 0.05, versus 5 mmol/L homocysteine-treated alone group.

We examined the effect of ACS6 on the release of Cyt-c in homocysteine-stimulated PC12 cells by Western blot analysis. As illustrated in Figure 4, treatment with homocysteine (5 mmol/L) for 24 h significantly promoted the release of Cyt-c in PC12 cells. However, the homocysteine-induced release of Cyt-c was significantly attenuated by pretreatment with ACS6 (16 μmol/L, 30 min). These results indicate that ACS6 prevents homocysteine-induced Cyt-c release.

### ACS6 reduces homocysteine-induced activation of caspase-3

**Figure 5:**
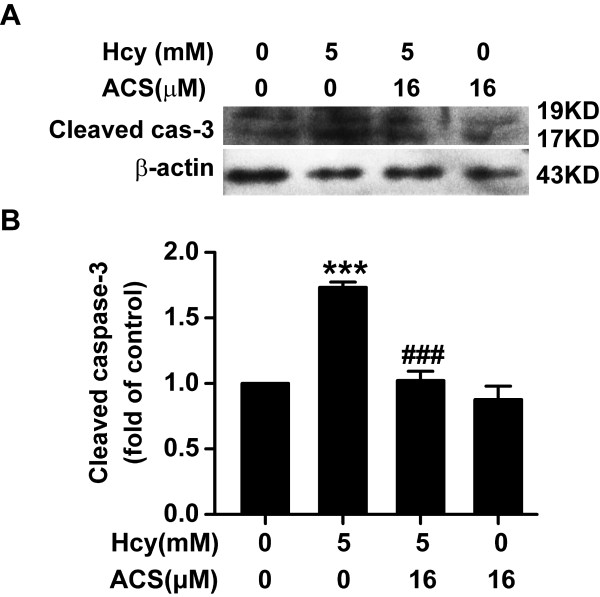
**Effects of ACS6 on the activation of caspase-3 in PC12 cells**. After pretreated with 16 μmol/L ACS6 for 30 min, PC12 cells were exposed to 5 mmol/L homocysteine (Hcy) for 24 h. The activation of caspase-3 in PC12 cells was analyzed by Western blot using an anti-cleaved caspase-3 antibody. Western blot images show representative results from three independent experiments. In all blots, staining for β-actin was used as a loading control. The level of cleaved caspase-3 expression obtained in each experimental condition was calculated as a fold of the control. Values are the mean ± SEM (*n *= 3). ***P <0.001, versus control; ^###^*P *< 0.001, versus 5 mmol/L homocysteine-treated alone group.

To investigate whether ACS6 modulates homocysteine-induced activation of caspase-3, the levels of caspase-3 activation were measured by Western blot analysis using anti-cleaved caspase-3 antibody. As illustrated in Figure 5, exposure to homocysteine (5 mmol/L, 24 h) significantly enhanced the expression of cleaved caspase-3 in PC12 cells. However, the homocysteine-induced enhancement of cleaved caspase-3 expression was significantly inhibited by pretreatment with ACS6 (16 μmol/L, 30 min). These results indicate that ACS6 blocks the homocysteine-induced activation of caspase-3.

### ACS6 attenuates homocysteine-induced accumulation of intracellular ROS

**Figure 6:**
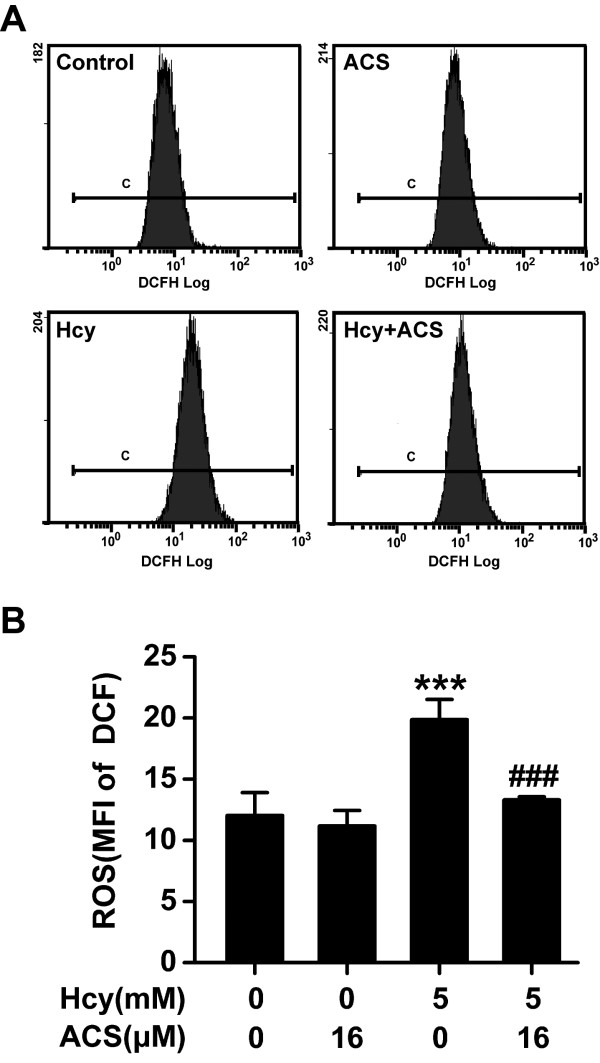
**Effects of ACS6 on homocysteine-exerted accumulation of intracellular ROS in PC12 cells**. After pretreated with 16 μmol/L ACS6 for 30 min, PC12 cells were exposed to 5 mmol/L homocysteine (Hcy) for 24 h and stained with DCFH-DA for 20 min. The changes of ROS in different treatment groups were quantified by fluorescent sorting FCM analysis (A, B). (A) Representative histogram of DCF-derived fluorescence in PC12 cells exposed to different treatments measured by FCM. (B) Quantitative analysis of the mean fluorescence intensity (MFI) of DCF measured by FCM. Values are the mean ± SEM (*n *= 3). ***P <0.001, versus control; ^###^*P *< 0.001, versus 5 mmol/L homocysteine- treated alone group.

As the cytotoxicity of homocysteine is mainly mediated by oxidative stress [10,39], we investigated the effect ACS6 on homocysteine-induced ROS formation by using DCFH-DA staining. Compared with non-treated control cells, the level of intracellular ROS was increased in PC12 cells treated with 5 mmol/L homocysteine for 24 h, as shown by the increase in the MFI of DCF quantified by FCM analysis (Figure 6A, B). However, when PC12 cells were co-treated with ACS6 (16 *μ*mol/L), the MFI of DCF (Figure 6A, B) in PC12 cells exposed to homocysteine (5 mmol/L, 24 h) were significantly decreased, suggesting that homocysteine-induced intracellular ROS accumulation is attenuated by ACS6. The cells treated with ACS6 (16 μmol/L) alone showed weak DCF fluorescence similar to that in the vehicle control (Figure 6).

### ACS6 prevents homocysteine-induced dissipation of Δψ

**Figure 7:**
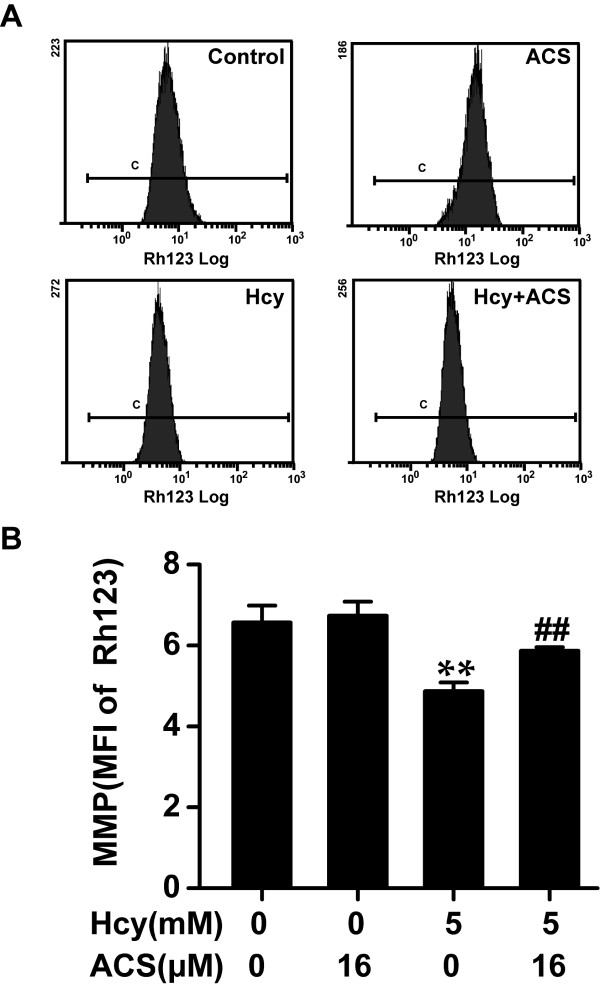
**Effects of ACS6 on homocysteine-induced loss of mitochondrial membrane potential (Δψ) in PC12 cells**. After pretreated with 16 μmol/L ACS6 for 30 min, PC12 cells were exposed to 5 mmol/L homocysteine (Hcy) for 24 h and stained with Rh123 for 20 min. The changes of Δψ in different treatment groups were quantified by fluorescent sorting FCM analysis. (A) Representative histogram of Rh123-derived fluorescence in PC12 cells exposed to different treatments measured by FCM. (B) Quantitative analysis of the mean fluorescence intensity (MFI) of Rh123 measured by FCM. Values are the mean ± SEM (*n *= 3). **P <0.01, versus control; ^##^*P *< 0.01, versus 5 mmol/L homocysteine-treated alone group.

Dissipation of Δψ is a critical event in the process of apoptosis [40]. To examine whether the anti-apoptotic effect of ACS6 involves preservation of Δψ, we used Rh123 staining to assess the level of Δψ in PC12 cells. After 24 h exposure to 5 mmol/L homocysteine, the Δψ was obviously reduced, as shown by the decrease in the MFI of Rh123 quantified by FCM analysis (Figure 7A, B), compared with non-treated control cells. Although ACS6 exposure alone (16 μmol/L) has no effect on Δψ of PC12 cells, the cells pretreated with ACS6 (16 μmol/L) for 30 min enhanced the intensity of Rh123 fluorescence in PC12 cells treated with homocysteine (5 mmol/L) for 24 h (Figure 7A, B). These results suggested that homocysteine-induced dissipation of Δψ is inhibited by ACS6.

### ACS6 reverses homocysteine-induced down-regulation of Bcl-2 expression

**Figure 8:**
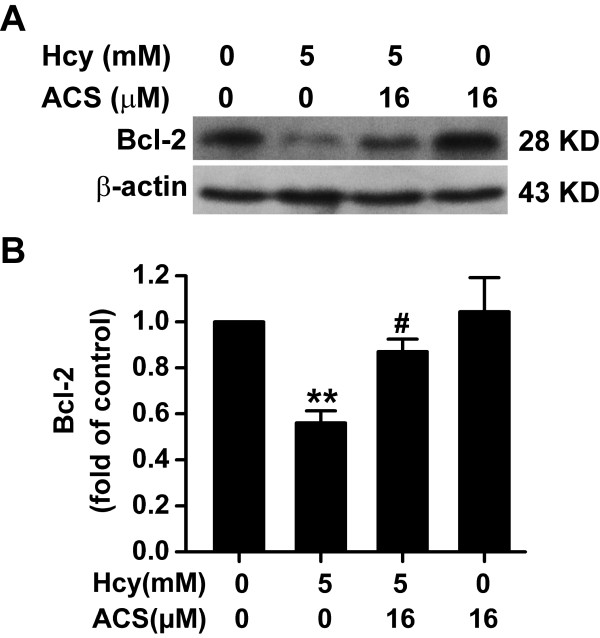
**Effects of ACS6 on the expression of Bcl-2 in PC12 cells**. After pretreated with 16 μmol/L ACS6 for 30 min, PC12 cells were exposed to 5 mmol/L homocysteine (Hcy) for 24 h. The levels of Bcl-2 expression in PC12 cells were determined by Western blot using an anti-Bcl-2 antibody. Western blot images show representative results from three independent experiments. In all blots, staining for β-actin was used as a loading control. The level of Bcl-2 expression obtained in each experimental condition was calculated as a fold of the control. Values are the mean ± SEM (*n *= 3). **P <0.01, versus control; ^#^*P *< 0.05, versus 5 mmol/L homocysteine-treated alone group.

Bcl-2 is an anti-apoptotic protein. To explore whether ACS6 modulates the effect of homocysteine on bcl-2 expression, the levels of bcl-2 were measured by Western blot analysis. As illustrated in Figure 8, exposure to homocysteine (5 mmol/L, 24 h) significantly reduced the expression of Blc-2 in PC12 cells. However, the homocysteine-induced decrease of Bcl-2 expression was significantly abolished by pretreatment with ACS6 (16 μmol/L, 30 min). These results indicate that ACS6 blocks the homocysteine-induced down-regulation of Bcl-2 expression.

## Discussion

Homocysteine is known to induce neurotoxicity and apoptosis [6,8] and cause oxidative damage [41]. Our previous results have demonstrated that homocysteine inhibits the activity and expression of CBS and the endogenous production of H_2_S in PC12 cells and this inhibitory effect contribute to the neurotoxicity of homocysteine [42]. We also found that H_2_S acts as a neuroprotectant counteracting an oxidative insult to neurons induced by homocysteine [31]. It has been shown that ACS6 inhibits the formation of superoxide by releasing H_2_S [32]. Therefore, we investigated whether ACS6 has the ability to attenuate the neurotoxicity of homocysteine.

PC12 cells, originally derived from a transplantable rat pheochromocytoma, are accepted as a model system for primary neuronal cells because of their ability to respond to nerve growth factor [43]. In the present study, we determined the neuroprotective effects of ACS6 on homocysteine neurotoxicity and the underlying mechanisms by studying PC12 cells. Similar to the findings by Linnebank *et al. *[9], we found that exposure of PC12 cells to homocysteine resulted in decrease of viability as well as increase of apoptotic cells. Furthermore, Cyt-c release and caspase-3 activation occurred in response to homocysteine in PC12 cells. These results indicated that homocysteine induces significant neurotoxicity and apoptosis in PC12 cell. Of important, the present work demonstrated that ACS6 not only attenuated the cytotoxicity and the apoptotic cells induced by homocysteine but also inhibited homocysteine-triggered Cyt-c release and caspase-3 activation in PC12 cells. This study is therefore the first to conclude that treatment with ACS6 blunts the apoptosis induced by homocysteine in PC12 cells.

It is well known that mitochondrial dysfunction is an important feature in apoptosis [44] as well as a prominent factor associated with cell death and some models of apoptosis [40]. Mitochondrial damage is consistent with intracellular ROS production and changes in Δψ during apoptosis [45]. Δψ has been shown to be involved in a variety of pathophysiological conditions, in particular for apoptosis [46,47]. ROS is responsible for the homocysteine-induced neurotoxicity [10,39]. Overproduction of ROS may result in mitochondrial dysfunction, causing Δψ loss and promoting Cyt-c release and caspase-3 activation, which ultimately cause cell apoptosis [48]. To investigate the mechanisms of the cytoprotective effect of ACS6 on homocysteine-induced apoptosis in PC12 cells, we examined its effects on the homocysteine-mediated changes in ROS and Δψ. The overproduction of ROS and dissipation of Δψ were significantly induced in homocysteine-exposed PC12 cells, while pretreatment with ACS6 prevented both phenomena. Our results suggested that the anti-apoptotic effect of ACS6 is associated with the preservation of mitochondrial function by blocking the dissipation of Δψ and the increase in ROS level.

It has been shown that Bcl-2 prevents apoptosis by regulating an antioxidant pathway [49]. Kane, *et al. *reported that bcl-2 inhibits neural death by reducing the generation of ROS [50]. Additionally, over-expression of bcl-2 increases stability of Δψ [51], and blocks cytochrome C release from mitochondria prior to mitochondrial membrane depolarization by preventing mitochondrial pore opening [52]. It is therefore established that the cytoprotective effects associated with decrease in ROS generation and stability of Δψ may be the results of over-expressed bcl-2. In the present study, we revealed that ACS6 blocked the down-regulation of bcl-2 induced by homocysteine. This finding implied that ACS6-induced up-regulation of bcl-2 expression may be involved in the protective actions of ACS6 against homocysteine-induced apoptosis and neurotoxicity.

In summary, our data for the first time demonstrated that ACS6 significantly limits the decrease in viability as well as the increase in apoptotic cells induced by homocysteine and prevents homocysteine-triggered Cyt-c release and caspase-3 activation. ACS6 not only blocks the loss of Δψ and overproduction of ROS caused by homocysteine but also upregulates the down-expression of Bcl-2 occurred in response to homocysteine. The findings support that ACS6 protects PC12 cells against homocysteine induced cytotoxicity and apoptosis and the underlying mechanism may involve preservation of mitochondrial function by inhibiting both loss of Δψ and accumulation of ROS as well as up-regulating the expression of bcl-2.

ACS6 is a putative H_2_S-donating derivative of sildenafil. It has been reported that H_2_S release from ACS6 is crucial for its biological action that inhibits the formation of superoxide [32]. In recent years, it has become clear that protects neurons from oxidative stress by increasing the levels of GSH [25,53] and attenuate myocardial ischemia-reperfusion injury by preserving mitochondrial function [54]. It is worthy to note that a previous study reported that H_2_S was detrimental in cerebral ischemia in rats [55]. This finding suggested that H_2_S is protective at concentrations that are equivalent to normal physiological concentrations, but is deleterious at supraphysiological concentrations in the brain. This is also supported by the data that excessive inhibition of H_2_S by AOAA led to detrimental effects in the brain [55]. Therefore, the ability of H_2_S to regulate cell viability may be concentration and time dependent. At low concentrations, as may occur in physiological conditions, cells remain unscathed by H_2_S, but, at high concentrations, as may occur in pathological states, a cytotoxic/proapoptotic effect becomes evident. To date, H_2_S-releasing "drugs" used in biological experiments has been largely restricted to simple sulfide salts, most commonly sodium hydrosulfide (NaHS), which releases H_2_S instantaneously in aqueous solution. However, the release of endogenous H_2_S from cells is likely to occur in lesser amounts and at a much slower rate than that from sulfide salts, and therefore NaHS may not mimic the biological effects of naturally produced H_2_S [56]. Muzaffar et al. reported that once taken up by cells, ACS6 would release H_2_S intracellularly and in a long-lasting controlled way [32]. Large amounts of H_2_S released over a short time frame by NaHS may trigger signaling pathways resulting in cell death, whereas this does not occur with the slower but sustained release of lower amounts of H_2_S from ACS6.

## Conclusions

In conclusion, the present findings clearly identify that ACS6, a novel H_2_S-releasing derivative, provides significant protection against homocysteine-induced neurotoxicity to PC12 cells by inhibiting both loss of Δψ and accumulation of ROS and up-regulating the expression of bcl-2. Based on the notion that elevated plasma homocysteine is a strong, independent risk factor of AD [13-17], our present study indicates that ASC6, or perhaps alternative related H_2_S-releasing compounds, could be worth to be further investigated in the study for their therapeutic uses as novel neuroprotectants for AD associated with homocysteine.

## Abbreviations

AD: Alzheimer's disease; CBS: cystathionine-β-synthetase; Cyt-c: cytochrome c; DCFH-DA 2',7':-dichlorfluorescein- diacetate; DCF 2',7':-dichlorfluorescein; FCM: flow cytometric; H_2_S: Hydrogen sulfide; MFI: mean fluorescent intensity; Δψ: mitochondrial membrane potential; PI: propidium iodide; Rh123: Rhodamine 123; ROS: reactive oxygen species

## Competing interests

Dr P Del Soldato is a shareholder of CTG Pharma, Milan, Italy. This company has patents on reagents used in this study. Professor Xiao-Qing Tang received a grant from CTG Pharma.

## Authors' contributions

XQT and RQC designed and developed this study. YKR, YYZ, HRF and CYW conducted experiments, analyzed data and coordinated the study. XQT wrote the manuscript. PDS and AS designed ASC6 and commented on the manuscript. All authors read and approved the final manuscript.
